# Exploring the Profile Contributions in *Meyerozyma guilliermondii* YB4 under Different NaCl Concentrations Using GC-MS Combined with GC-IMS and an Electronic Nose

**DOI:** 10.3390/molecules28196979

**Published:** 2023-10-08

**Authors:** Yiling Xiong, Ju Guan, Baozhu Wu, Tianyang Wang, Yuwen Yi, Wanting Tang, Kaixian Zhu, Jing Deng, Huachang Wu

**Affiliations:** 1 College of Food and Biological Engineering, Chengdu University, Chengdu 610106, China; xiongyiling@stu.cdu.edu.cn (Y.X.);; 2 Cuisine Science Key Laboratory of Sichuan Province, Sichuan Tourism University, Chengdu 610100, China

**Keywords:** aroma-producing yeast, NaCl, flavor profile, GC-MS, GC-IMS, E-nose

## Abstract

Using *Meyerozyma guilliermondii* YB4, which was isolated and screened from southern Sichuan pickles in the laboratory, as the experimental group, we investigated the changes in growth, total ester content, and volatile flavor substances of *M. guilliermondii* YB4 under different NaCl concentrations. The growth of *M. guilliermondii* YB4 was found to be inhibited by NaCl, and the degree of inhibition increased at higher NaCl concentrations. Additionally, the total ester content of the control group (CK) was significantly lower compared to the other groups (*p* < 0.05). The application of NaCl also resulted in distinct changes in the volatile profile of YB4, as evidenced by E-nose results. Gas chromatography-mass spectrometry (GC-MS) and gas chromatography-ion mobility spectrometry (GC-IMS) were employed to analyze the volatile compounds. A total of 148 and 86 volatiles were detected and identified using GC-MS and GC-IMS, respectively. Differential volatiles among the various NaCl concentrations in YB4 were determined by a variable importance in projection (VIP) analysis in partial least squares-discriminant analysis (PLS-DA). These differentially expressed volatiles were further confirmed by their relative odor activity value (ROAV) and odor description. Ten key contributing volatiles were identified, including ethanol, 1-pentanol, nonanal, octanal, isoamyl acetate, palmitic acid ethyl ester, acrolein, ethyl isobutanoate, prop-1-ene-3,3’-thiobis, and 2-acetylpyrazine. This study provides insights into the specificities and contributions of volatiles in YB4 under different NaCl concentrations. These findings offer valuable information for the development of aroma-producing yeast agents and the subsequent enhancement in the flavor of southern Sichuan pickles.

## 1. Introduction

Southern Sichuan pickles, represented mainly by Neijiang kohlrabi and Yibin sprout, are a popular traditional fermented food in southwest China. They undergo natural fermentation in a high concentration salt environment, followed by protein decomposition, desalination, and dehydration processes, resulting in a characteristic fresh and crisp texture with salty and spicy flavors [1]. This pickle is a nutrient-rich food, containing high levels of protein, vitamins, minerals, and other essential nutrients [2]. Moreover, southern Sichuan pickles undergo a fermentation process that enhances their texture to become crisp and tender while developing a rich aroma and delightful taste. As a result of these qualities, they have gained immense popularity as a favored accompaniment throughout China. The microbial system involved in the natural fermentation of traditional pickles is complex, consisting of lactic acid bacteria (LAB) and yeast as dominant microorganisms. LAB and yeast work together to create the unique flavor of pickles [3]. LAB are responsible for the production of lactic acid and bacteriocin, which facilitate the rapid and safe acidification of brines [4]. Meanwhile, yeast, particularly aroma-producing yeast, plays a crucial role in shaping the flavor profile of southern Sichuan pickles. Aroma-producing yeast possesses the ability to esterify acids and alcohols, leading to the formation of various flavorful compounds with ester aromas [5]. Inoculating aromatic yeast into Daqu, the fermentation starter used in liquor production, can modulate the metabolic activity of the microbial community and increase the content of ethyl caproate, thereby enhancing the fruity aroma of the liquor [6]. However, despite its significance in the formation of fermented pickle flavors, there have been limited studies investigating how aromatic yeast specifically influences the flavor of fermented pickles.

The microbial ecology during the natural fermentation process of southern Sichuan pickles is complex and influenced by various factors. These factors include intrinsic factors such as the diffusion of sugars, amino acids, organic acids, and more; the natural microorganisms present in the pickles and their interactions; and extrinsic factors like the addition of salt, humidity, and temperature [7,8]. Salt (NaCl) is a crucial ingredient in vegetable fermentation that affects sensory characteristics and safety. Its concentration can influence the microbial structure and metabolic activity during vegetable fermentation, thereby impacting the flavor profile of the final product. For instance, a study conducted by Chun et al. demonstrated that fermenting Korean dajiang with different salts had a significant impact on the flavor profile of dajiang paste due to its influence on microbial metabolic activity. Specifically, it was found that low-salt (9%) dajiang exhibited a higher abundance of alcohol and ester volatile compounds [9]. However, despite its importance in the fermentation flavor of southern Sichuan pickles, there have been few studies on the effects of NaCl on the microorganisms associated with these pickles. To address these gaps, several aroma-producing yeast strains have been isolated from southern Sichuan pickles [10], which were identified as YB4 (*M. guilliermondii*), YB18 (*D. hansenii*), and YC14 (*C. parapsilosis*) via ITS rDNA. Notably, *M. guilliermondii* YB4 demonstrated the broadest salt tolerance, although its aroma production ability remains unclear.

Gas chromatography-mass spectrometry (GC-MS) and electronic noses (E-noses) have become commonly employed techniques for analyzing volatile compounds in fermented food. GC-MS enables qualitative and quantitative analyses of volatile flavor substances with high sensitivity [11], combining the safety, accuracy, and excellent separation ability of headspace solid-phase microextraction and gas chromatography, as well as the identification capabilities of mass spectrometry [12]. Secondly, E-noses utilize chemical sensors and pattern recognition systems to rapidly detect and differentiate overall volatile substances in food [13]. However, both GC-MS and E-noses have their limitations. The presence of isomers in mass spectrometry can affect the detection and separation in GC-MS analysis [14]. E-noses are only able to detect and classify volatile compounds in samples to a certain extent. Fortunately, gas chromatography-ion mobility spectrometry (GC-IMS) can address these shortcomings. By combining the advantages of gas chromatography (GC) and ion mobility spectrometry (IMS), such as their high resolution and sensitivity, GC-IMS offers a solution for qualitative and quantitative analyses of trace volatile organic compounds (VOCs), especially for isomer analysis [15]. It has found extensive applications in the analysis of volatile compounds in fermented foods, including wines [16], Suancai [17], and chili sauce [18]. Recently, there has been growing interest in studying volatile flavor substances in foods through the combination of various techniques. For instance, Zhang et al. investigated the changes in quality and flavor profiles of different fresh strawberry juices during cold storage using GC-IMS, GC-MS, and an E-nose [19]. The integration of multiple technologies provides more comprehensive, reliable, and scientific information about food aromas.

The primary purpose of this study was to explore the effects of *M. guilliermondii* YB4, which was isolated and screened from pickles in southern Sichuan in the early stage of the experiment, this was used as the experimental group to explore the changes in total ester production ability and volatile flavor substances of YB4 under five NaCl concentrations of 0% (CK), 6% (APYA), 9% (APYB), 12% (APYC) and 15% (APYD). An E-nose and GC-MS combined with GC-IMS were selected to analyze the flavor compounds and determine the critical odor active compounds. The results will provide a detailed theoretical basis of the preparation of aroma-producing yeast viable agents and the subsequent development of southern Sichuan pickles.

## 2. Results and Discussion

### 2.1. Determination of YB4 Growth Curve and Total Esters with Different Salt Concentrations

**Figure 1:**
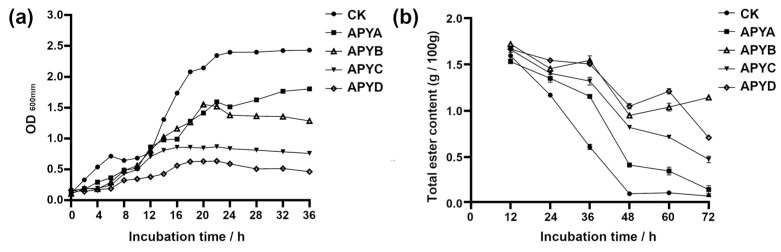
(**a**) Determination of Y4 growth curves at different NaCl concentrations; (**b**) changes in total esters with fermentation time.

Based on the YB4 curve (Figure 1a), it can be observed that the addition of NaCl to the fermentation broth resulted in a notable slowdown in the growth of YB4, particularly in terms of APYD. This phenomenon suggests that NaCl exerts inhibitory effects on the growth of YB4, and the degree of inhibition intensifies with increasing NaCl concentrations. The total ester content serves as a direct measure of the overall quantity of esters produced by aroma-producing yeasts, which is a crucial indicator of aroma and flavor in pickles. Figure 1b demonstrates a decrease in the total ester content in the fermentation broth of YB4 as the incubation time increases, implying that a substantial portion of the total ester has undergone conversion into volatile esters. A significantly lower total ester content was observed in the CK group at 24–72 h compared to the other groups (*p*-value < 0.05), potentially indicating an enrichment of flavor compounds facilitated by NaCl. Moreover, the enrichment effect was more pronounced with higher concentrations of NaCl relative to lower concentrations. The observed increase in total ester content in APYD at 48–60 h and APYB at 48–72 h may be attributed to the fact that the total ester content produced by YB4 surpasses the amount consumed during the conversion into volatile esters, resulting in an overall accumulation of total esters.

### 2.2. E-Nose Analysis

**Figure 2:**
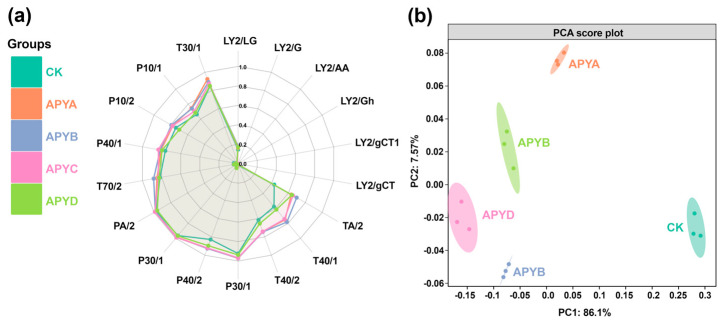
(**a**) Radar chart of scores obtained by different sensors of YB4 with different NaCl concentrations. (**b**) PCA model built on sensors scores.

The overall volatile flavor of YB4 after fermentation, as well as the composition of substances, was analyzed using an E-nose, as depicted in Figure 2a. The 18 coordinate axes in the figure represent 18 sensors individually, with the size of each axis indicating the sensor’s sensitivity. Based on the observations depicted in Figure 2a, it can be inferred that none of the samples exhibited significant responses from sensors LY2/G, LY2/AA, LY2/Gh, LY2/gCT1, and LY2/GT, suggesting a minimal production of nitrogen oxides, amine compounds, sulfides, and acetone during YB4 fermentation. Conversely, APYB and APYC demonstrated higher response values for sensors TA/2, T40/1, T40/2, P30/2, P40/2, P30/1, PA/2, T70/2, P40/1, P10/2, P10/1, and T30/1 compared to other samples. These results indicate a significant presence of alcohols, aromatic compounds, hydrocarbons, and ammonia in APYB and APYC relative to the other samples.

To assess the overall differences among various sample treatments, principal component analysis (PCA) was utilized as a multivariate statistical tool. The results are illustrated in Figure 2b, where PC1 and PC2 account for 86.1% and 7.57% of the total variance, respectively, cumulatively representing 93.67% of the total variance. This suggests that these two principal components effectively capture all the information characteristics of YB4. Notably, CK appears significantly distant from the other samples, indicating a notable difference in the flavor of YB4 upon the addition of NaCl. Conversely, APYA, APYB, and APYC are located close to each other, indicating similar aromas of YB4 within the 6% to 12% NaCl concentration range.

### 2.3. GC-MS Analysis

**Figure 3:**
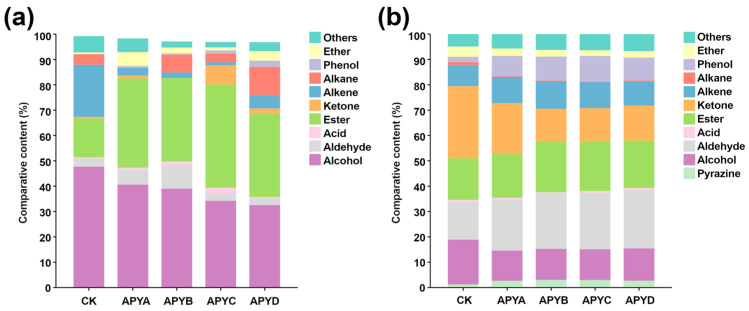
Relative percentage of each category volatiles in YB4 with different NaCl concentrations by GC-MS (**a**) and GC-IMS (**b**).

GC-MS was employed to analyze the volatile profiles of YB4 under different NaCl concentrations. A comprehensive total of 147 volatiles were detected and identified, with CK containing 53 volatiles, APYA containing 44 volatiles, APYB containing 45 volatiles, APYC containing 41 volatiles, and APYD containing 43 volatiles. The detailed results of these volatiles can be found in Table S1, including 29 alcohols, 11 aldehydes, 7 acids, 30 esters, 8 ketones, 22 alkenes, 17 alkanes, 2 phenols, 6 ethers, and 15 others. Figure 3a depicts the variations in the content of volatile profiles across the five NaCl concentrations of YB4 as determined by GC-MS. Notably, esters and alcohols exhibited the highest diversity and content among the volatile aroma substances detected. As illustrated in Figure 3a, the alcohol content gradually decreased with increasing salt concentration, while the ester content initially increased and then decreased. Alcohols are known precursors of aromatic esters, which are a significant source of esters in aromatic yeasts. Alcohols contribute floral and fruity aromas to foods and are primarily produced through microbial-induced sugar metabolism, redox reactions of unsaturated aldehydes or ketones, and amino acid metabolism [20,21]. Esters, on the other hand, represent a notable category of sensory-active compounds that impart characteristic fruity and floral aromas while mitigating the intensity of unpleasant odors in final products [22]. They are typically formed through esterification and microbial metabolism [21,23].

Overall, significant variations in the content of various volatiles were observed across the five NaCl concentrations of YB4. The concentration of all volatile compounds was highest in CK, while it was lowest in APYD, indicating that NaCl inhibited flavor formation in YB4. In CK (Table S1), ethanol, isobutanol, and 2-methylbutanol exhibited higher contents compared to other volatiles. These compounds possess alcohol- and malt-like odor properties and likely contribute significantly to the overall aroma of CK. Interestingly, ethanol and 2-methylbutanol were also the dominant volatiles in APYA, APYB, and APYC. However, their contents were significantly lower than those in CK (Table S1). In APYD, hexadecanoic acid ethyl ester, 2-methylbutanol, ethyl L-alaninate hydrochloride, and 2,3-butanediol exhibited higher contents compared to most other volatiles. These compounds possess alcohol- and fruit-like odors, respectively.

### 2.4. GC-IMS Analysis

**Figure 4:**
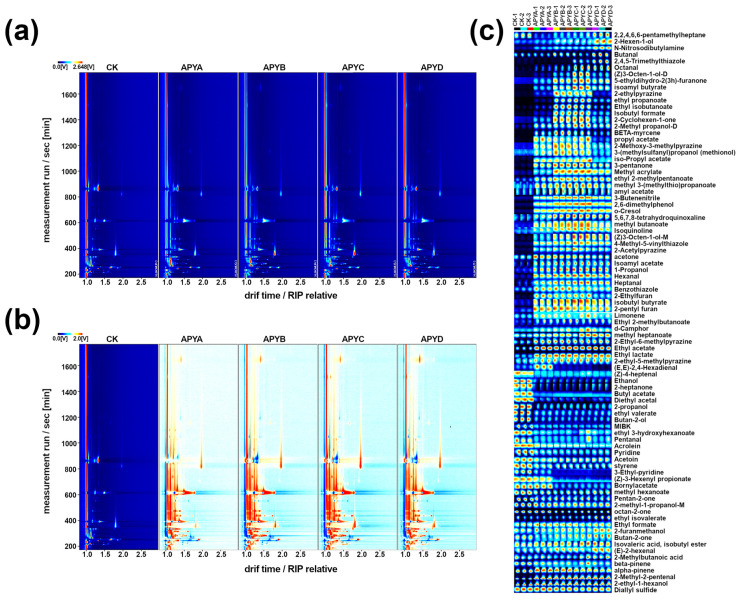
GC-IMS plots of volatile flavor substances in YB4 with different NaCl concentrations: (**a**) 2D topographic images; (**b**) 2D topographic images with differential comparison mode; (**c**) the Gallery Plot of volatile flavor substances.

The volatile profiles of *M. guilliermondii* YB4 under different NaCl concentrations were further analyzed using GC-IMS. The representative detection fingerprints are presented in Figure 4, where the X, Y, and Z axes correspond to the ion drift time, retention time, and peak intensity, respectively [24]. Each point in the fingerprint represents a volatile compound in the sample, with the color indicating the intensity of the peak. Darker colors indicate higher peak intensities. As shown in Figure 4, the volatile components of YB4 at five NaCl concentrations exhibited significant separation. Most peaks had retention times of 200–1600 s and drift times of 1.0–2.5 s. The fingerprints of the five types of YB4 displayed notable differences, particularly in the CK samples where signal intensities varied (Figure 4c). Moreover, APYB and APYC exhibited a higher abundance of volatile compounds. The qualitative results and relative contents of all volatiles in YB4 samples at the five NaCl concentrations are listed in Table S2. A total of 86 volatiles were detected and identified, including 13 alcohols, 9 aldehydes, 27 esters, 10 ketones, 5 alkenes, 2 alkanes, 2 phenols, 1 acid, 1 ether, 5 pyrazines, and 11 others. These categories differed from the results obtained using GC-MS. Additionally, GC-IMS allowed for the accurate detection and identification of some volatile monomers and dimers, which was not possible with GC-MS [25].

Figure 3b illustrates the variations in the content of volatile profiles at five NaCl concentrations for YB4 as determined by GC-IMS. These profile differences were distinct from those observed in Figure 3a. Considering the differences in detection principles, GC-MS and GC-IMS complement each other. Their combination provides a more comprehensive and unbiased depiction of the volatile profiles. As depicted in Figure 3b, aldehydes, esters, and ketones were the predominant flavor profiles in YB4 under different NaCl concentrations. With increasing NaCl concentration, the alcohol content gradually decreased, consistent with the results obtained from GC-MS, while the aldehyde content increased gradually. Aldehydes are primarily formed through the oxidation and degradation of lipids, imparting pleasant grassy, malty, and fruity flavors and aromas, which strongly influence the overall sensory profile of food due to their low threshold values. They are mainly derived from the oxidative cleavage of unsaturated fatty acids and the Strecker degradation of amino acids [26]. Additionally, microbial aminotransferases can convert free amino acids (FAAs) into α-ketoacids, which are further degraded into the corresponding aldehydes by various decarboxylases during fermentation [27]. The aldehyde with the highest content in YB4 was 2-methyl-2-pentenal, which serves as a crucial indicator of lipid oxidation [28]. According to Choi et al. [29], hexanal and 2-methyl-2-pentenal are produced as a result of residual enzyme activity during the drying process of onion substrates. Consequently, it can be inferred that the variations in levels of hexanal and 2-methyl-2-pentenal in YB4 under different NaCl concentrations are likely due to the varying activities of residual enzymes. These variations further contribute to the diverse degrees of lipid oxidation observed in the final product. Interestingly, GC-IMS detected the presence of high levels of ketones, particularly in CK samples of YB4. Ketones are mainly formed through the oxidation of unsaturated fatty acids and the degradation of amino acids. Most ketones have unique light fruity aromas [30]. Octan-2-one was the most abundant ketone in all YB4 samples, reported as the most volatile flavor substance in fresh shiitake mushrooms, associated with creamy and fruity flavors [31]. Its concentration significantly decreased with increasing NaCl concentrations for YB4.

### 2.5. Differential Volatile Compounds

**Figure 5:**
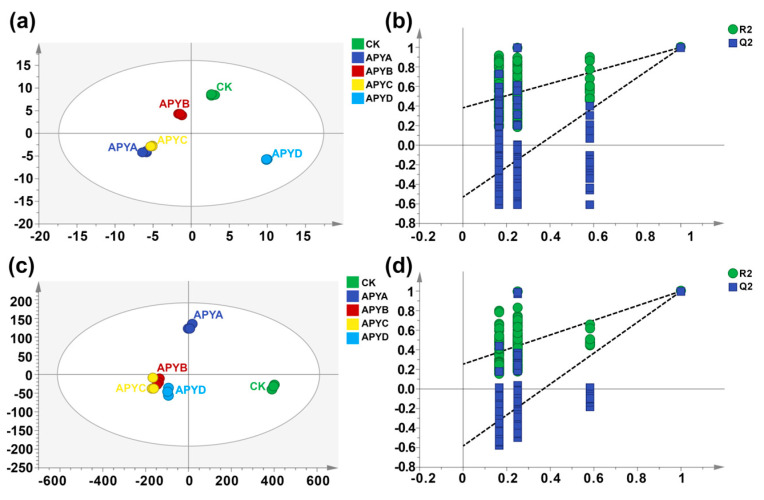
The PLS-DA results of YB4 with different NaCl concentrations using GC-MS and GC-IMS. (**a**) PLS-DA score plots via GC-MS; (**b**) PLS-DA scores plots via GC-IMS; (**c**) cross-validation plot after 200 permutation tests for GC-MS; (**d**) cross-validation plot after 200 permutation tests for GC-IMS.

**Figure 6:**
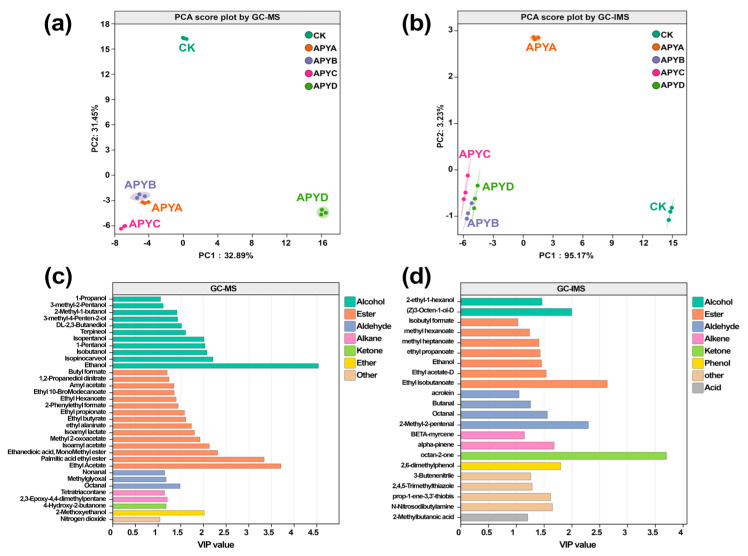
The PCA score plots of the volatile profiles identified by (**a**) GC-MS and (**b**) GC-IMS. The VIP values of the PLS-DA models for the candidate differential volatiles among YB4 with different NaCl concentrations were determined by (**c**) GC-MS and (**d**) GC-IMS.

To examine the variations in volatiles among YB4 samples with different NaCl concentrations, a principal component analysis (PCA) was conducted using the data of aroma compounds detected by both GC-MS and GC-IMS. The obtained score plots are presented in Figure 5a,b. As depicted in Figure 5, YB4 samples of the same aroma type clustered together, while YB4 samples of different aroma types exhibited distinct separations. This observation suggests significant differences in volatile profiles among YB4 samples with varying NaCl concentrations, as detected by both GC-MS and GC-IMS. In order to evaluate the contribution of each volatile compound to the classification, the variable importance in the projection (VIP) in the supervised PLS-DA model was calculated. Volatiles with a VIP value greater than 1 were deemed as significant contributors to the classification [32]. Thus, supervised PLS-DA analyses were performed on the volatile profiles measured by GC-MS and GC-IMS, respectively. The results are displayed in Figure 5a,b. After conducting the permutation test, it was found that the constructed PLS-DA models exhibited satisfactory performance without overfitting or underfitting. Based on the VIP values derived from the PLS-DA models (Figure 6c,d), a total of 35 and 22 volatiles were identified as candidate differential compounds by GC-MS and GC-IMS, respectively. Among these, the 35 volatiles determined by GC-MS consisted of 11 alcohols, 15 esters, 3 aldehydes, 1 ketone, 2 alkanes, 1 ether, and 1 other compound.

For GC-IMS, these 22 volatiles included 2 alcohols, 7 esters, 4 aldehydes, 2 alkenes, 1 ketone, 1 phenol, 1 acid, and 4 others. Nevertheless, these differential volatiles should be further validated to ascertain their specific contribution to the overall aroma of YB4. Regional classification performed using PLS-DA showed that GC-MS could identify more differential compounds due to different detection principles, while GC-IMS was better suited for classifying YB4 under varying NaCl concentrations in comparison to GC-MS. This may be because different NaCl concentrations have greater effects on small molecule metabolites in YB4. Therefore, a combined approach involving GC-MS and GC-IMS for the characterization and classification of volatile organic compounds in YB4, combined with appropriate multivariate analysis, has the potential to be used as a non-destructive method to evaluate the flavor of aroma-producing yeast.

### 2.6. ROAV Calculations for Volatile Compounds in YB4 

To assess the contributions of compounds to the flavor of food, the Relative Odor Activity Value (ROAV) can be calculated based on the relative content of each compound and a predefined threshold value [33]. The contribution of individual volatiles to the overall aroma of YB4 depends on the ROAV. Table 1 shows that the nonanal content in GC-MS results was relatively higher and its threshold was low, and thus its contribution to the overall flavor of YB4 was greatest. Thus, ROAVstan (=100) was defined by the nonanal content. Similarly, ROAVstan was defined in the GC-IMS result by the content of ethyl 2-methylpentanoate (=100). Then, the ROAVs of other volatile flavor substances were calculated. The statistics of ROAV > 1 in the volatile profiles measured by GC-MS and GC-IMS are presented in Table 1 and Table 2, respectively. A total of 25 volatiles out of 143 were selected for GC-MS analysis, including nonanal, 2-methyl-1-propanol, 3-methyl-1-butanol, ethanol, octanal, etc. The volatile flavor profile of CK was determined by GC-MS analysis (Table 1), primarily consisting of nonanal, 2-methyl-butanal, ethyl isobutyrate, butyl acrylate, 2-methyl-1-propanol, and other compounds. In contrast, the dominant volatile flavor components in APYA were identified as ethyl isobutyrate, 3-methyl-1-butanol, and other compounds. Similarly, benzeneacetaldehyde, 2-methyl-1-propanol, eucalyptol, and other compounds were found to be the main contributors to the volatile flavor profile of APYB. On the other hand, the primary volatile flavor components in APYC included ethyl isobutyrate, 3-methyl-1-butanol, 2-methyl-1-propanol, and other compounds. Finally, the major volatile flavor constituents in APYD were identified as 3-methyl-1-butanol, butyl acrylate, eucalyptol, and other compounds. In general, the predominant volatile flavor compounds in YB4 were derived from alcohols, aldehydes, and esters. However, variations in flavor were observed at different NaCl concentrations. In the presence of high concentrations of NaCl, the contribution of aldehydes to YB4 flavor significantly decreased, whereas the contribution of alcohols and ethers increased. This phenomenon can be attributed to the oxidation of aldehydes into acids and their reduction into alcohols, thereby attenuating the impact of aldehydes [34]. 

For GC-IMS, 10 volatiles out of 86 were selected, including ethyl 2-methylpentanoate, acrolein, ethyl valerate, ethyl 2-methylbutanoate, etc. Notably, the contribution of 2,6-dimethylphenol increased in all the remaining samples’ flavor profiles compared to CK, implying YB4 has a coffee aroma. A possible explanation for this phenomenon could be that the presence of NaCl hinders the process of phenol hydrolysis. Phenols are the most important antioxidant in kohlrabi [1] and grapes [35]. Overall, 25 volatiles detected by GC-MS and 10 volatiles detected by GC-IMS were identified as contributing compounds in the YB4 samples with varying NaCl concentrations, as their ROAV values exceeded 1. However, it is worth noting that the thresholds for some of these volatiles remain unknown. To comprehensively quantify and evaluate the contribution of each volatile to yeast-derived aromas, further systematic investigations are required to explore additional volatiles in water, along with their respective thresholds and corresponding ROAV values.

### 2.7. Contributing Volatiles in YB4 with Different NaCl Concentration

**Figure 7:**
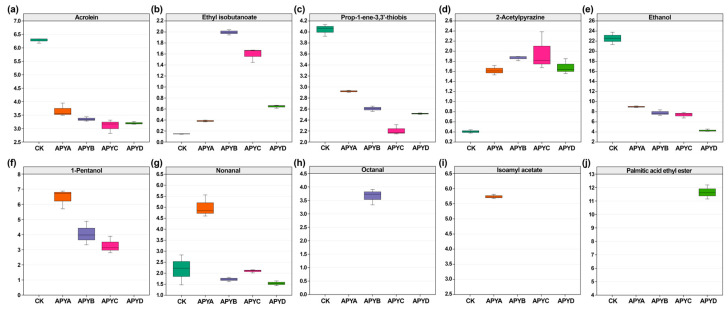
Boxplots of the contributing volatiles in YB4 at different NaCl concentrations: (**a**) acrolein, (**b**) ethyl isobutanoate, (**c**) prop-1-ene-3,3’-thiobis, (**d**) 2-acetylpyrazine, (**e**) ethanol, (**f**) 1-pentanol, (**g**) nonanal, (**h**) octanal, (**i**) isoamyl acetate, and (**j**) palmitic acid and ethyl ester.

The differential volatiles, identified using VIP > 1 from the PLS-DA models, underwent subsequent analyses. To determine the key contributing volatiles in YB4 at five different NaCl concentrations, integration of the ROAV and odor description was performed for each individual differential volatile. This process is illustrated in Figure 7. Ten key contributing volatiles were identified across all samples, including ethanol, 1-pentanol, nonanal, octanal, isoamyl acetate, palmitic acid, and ethyl ester, as measured by GC-MS, and acrolein, ethyl isobutanoate, prop-1-ene-3,3’-thiobis, and 2-acetylpyrazine, as measured by GC-IMS. As shown in Figure 6, the contents of acrolein, prop-1-ene-3,3′-thiobis, and ethanol in CK were significantly (*p* < 0.05) higher than those in other samples, meanwhile 1-pentanol, nonanal, and isoamyl acetate contents in APYA were significantly higher than that in other samples. The combination of these compounds provides an aroma-producing yeast with floral, fruity, alcoholic, and spicy fragrances.

Ethanol exhibits a significant role in enhancing aroma through synergistic effects with other flavor compounds, which can be converted into esters and other aromatic components [36,37]. Studies have shown that aroma-producing yeasts can convert ethanol into aldehydes, esters, and other substances [6]. According to Tables S1 and S2, ethanol was detected by both GC-MS and GC-IMS, and it contents decreased significantly with the increase in salt concentration (*p* < 0.05). The reason for this may be that ethanol is consumed in large quantities to produce esters [38]. 1-Pentanol, which has sweet and fusel odor, is produced by the degradation of amino acids through the Strecker reaction to produce corresponding aldehydes which are then reduced [24]. As shown in Figure 7, the content of 1-pentanol was the highest in APYB, but not detected in CK and APYD. The reason for this may be that high salt concentrations inhibited the metabolism of yeast and lipid oxidation. Secondly, previous studies have shown that salt is the key to sustained lipid oxidation [39].

As for esters, ethyl esters constituted the largest group of esters. Short-chain fatty acid ethyl esters (SCFAEE) and medium-chain fatty acid ethyl esters (MCFAEE) exhibit lower odor thresholds, predominantly characterized by fruity and floral aromas such as ethyl acetate, ethyl butyrate, and ethyl isobutanoate. On the other hand, long-chain fatty acid ethyl esters (LCFAEE) primarily present an ether or wax aroma profile with compounds like ethyl valerate and palmitic acid ethyl ester. Palmitic acid ethyl ester has a fatty, fruity, and rancid odor [40] and was detected only in APYD. Wang et al. found that palmitic acid ethyl ester was one of the main LCFA ethyl esters in red beancurd, giving red beancurd a fatty and oily odor [37]. Ethyl isobutanoate exhibits a sweet and alcoholic odor, and was the highest in APYB, where it was possibly formed by the oxidation and metabolism of fatty acids or amino acid metabolism [27]. The presence of these esters is related to the metabolism of lipid by yeast, which is involved in the formation of free fatty acids to facilitate the synthesis of corresponding ethyl esters with alcohols [41]. Isoamyl acetate was recognized as an important flavor compound that contributes to the fruity flavor of fruit wine [42]. Yang et al. found that the content of isoamyl acetate increased significantly during the aging process of Chinese rice wine (Huangjiu) [43]. However, the addition of a certain amount of NaCl resulted in all of these esters exhibiting a high content in YB4.

Acrolein, a compound with a pungent odor, is primarily formed through microbial metabolism. In a study conducted by Zhang et al., it was discovered that acrolein acts as the major differentiating flavor component in both traditional and modern fermented Xuecai [44]. As shown in Figure 7, the concentration of acrolein is highest in the CK sample, but significantly decreases (*p* < 0.05) as the NaCl concentration increases. This decline can be attributed to the inhibitory effect of NaCl on the metabolism of YB4. Nonanal and octanal, which possess a fatty and citrusy aroma, are derived from the beta oxidation of oleic acid. The decomposition of n-9 polyunsaturated fatty acids containing oleic acid results in the production of octanal and nonanal [45]. 2-Acetylpyrazine, known for its baked and nutty aroma, is formed through the condensation of two α-amino ketone molecules via Strecker degradation [46]. In Figure 7, the levels of 2-acetylpyrazine in the CK sample are significantly lower (*p* < 0.05) compared to the other samples, indicating that the addition of NaCl contributes to the production of 2-acetylpyrazine. Ether compounds generally yield pleasant aromas [39]. Prop-1-ene-3,3’-thiobis has the aroma of garlic and pepper, and it serves as a key aroma-active substance in black garlic. It can also be used as an indicator to detect the flavor profile of black garlic [47].

In summary, our study has provided preliminary identification of the key volatiles that play a significant role in differentiating YB4 samples with varying NaCl concentrations (Figure 7). However, it is important to note that additional research is required to validate and refine the aforementioned findings. On the one hand, the volatiles in YB4 are complex to the whole aroma of pickles in southern Sichuan, and there may be synergistic or antagonistic effects. Therefore, it is necessary to further explore the comprehensive effect of YB4 on the flavor of pickles in southern Sichuan. On the other hand, due to the availability of thresholds for various volatiles in YB4, some other possible key contribution volatiles may not be adequately demonstrated.

## 3. Materials and Methods

### 3.1. Bacterial Strains and Growth Conditions

*M. guilliermondii* YB4 isolated from south Sichuan pickles (Sichuan, China) was used in pickles to produce flavor in the fermentation process and was preserved in the Culinary Science Key Laboratory of Sichuan Province, Sichuan Tourism University. *M. guilliermondii* YB4 strains were suspended in potato dextrose agar medium (PDA, 200 g/L potato, 20 g/L glucose, 15–20 g/L agar) with 25% glycerol and preserved at −60 °C. The preserved strain was streaked in yeast peptone dextrose medium (YPD, 10 g/L yeast extract, 20 g/L peptone, 20 g/L dextrose, 20 g agar) and cultured in a constant temperature incubator at 28 °C for 2–3 days to activate the strain. The activated strain was inoculated with 50 mL yeast extract peptone dextrose medium (YEPD, 10 g/L yeast extrac, 20 g/L peptone, 20 g dextrose) and cultured at 28 °C and 150 rpm for 20 h, and was then used for subsequent experiments.

### 3.2. Reagents and Standards

YPD, YEPD, and PDA media were purchased from Chengdu Jinshan Chemical Reagent Co., Ltd. (Chengdu, China). Sodium hydroxide, phenolphthalein, agar, glucose, yeast extract, and NaCl were purchased from Beijing Aoboxing Biotechnology Co., Ltd. (Beijing, China).

### 3.3. Determination of Growth Curves

The growth curve was determined according to the absorbance value measured via an ultraviolet spectrophotometer (Lebo Tech Instrument Co., Ltd., Beijing, China). Samples were taken (5 mL) every 2 h to measure the absorbance value (A600 nm). The culture conditions of the samples were set at 28 °C, respectively, for 36 h (150 rpm). We repeated the measurement three times for YB4 at each NaCl concentration.

### 3.4. Detection of Total Esters

The prepared bacterial suspension was inoculated into 200 mL PDA liquid medium containing 0%, 6%, 9%, 12%, and 15% NaCl at a ratio of 5%. The PDA liquid medium with 0% concentration of NaCl was used as the blank group, and cultured at 28 °C and 150 rpm for 12, 24, 30, 36, 48, 60, and 72 h.

Determination of total ester: Four milliliters of fermentation broth was diluted 10 times and distilled at 100 °C for 30 min, and then the total ester content in the distillate was determined by the reflux saponification method. The contents of total ester were determined according to Jiang et al. [48].

### 3.5. Volatile Compound Analysis

#### 3.5.1. E-Nose Analysis

A Fox 4000 Sensory Array Fingerprint Analyzer (Alpha M.O.S., Toulouse, France) equipped with an array of 18 different metal oxide sensors and pattern recognition software for data recording and analysis was employed. The analysis involved placing 1 mL of the yeast fermentation broth sample into a sample vial and sealing it, followed by heating at 70 °C in an air bath to generate headspace for 300 s to ensure that the headspace volatiles were equilibrated at the start of the experiment. The carrier gas used was clean dry air, with a flow rate of 150 mL/min, injection speed of 500 μL/s, and injection period of 1 s. Odor data were recorded every second for 120 s. Each sample was analyzed in eight parallel trials.

#### 3.5.2. Volatile Compounds Detected by GC-MS

Specifically, 3 mL of yeast fermentation broth with different concentrations of NaCl cultured for 72 h was placed in a 15 mL headspace flask. A rotor was then added to facilitate complete evaporation of the volatiles. A magnetic stirring device (PC-420D, Corning Inc., Corning, NY, USA) maintained a temperature of 120 °C and rotated at a speed of 1.5 r/s for an equilibrium time of 600 s. Subsequently, the extraction head (250 °C, 600 s) was inserted into the sample bottle for extraction over a duration of 7200 s before being introduced into the GC-MS instrument.

GC-MS analysis was performed on a PerkinElmer SQ680 (Perkin Elmer Instruments Inc., Waltham, MA, USA). Volatiles were separated using an Elite-5MS (30 m × 0.25 mm × 0.25 μm, Perkin Elmer Instruments Inc., Waltham, MA, USA). Helium (>99.999%) was used as the carrier gas at a constant flow rate of 1 mL/min. The oven temperature was initially set at 40 °C for 1 min, followed by a linear ramp up to 170 °C at a rate of 5 °C/min. After maintaining the temperature at 170 °C for 1 min, it was further increased to 250 °C at a rate of 15 °C/min. The final temperature of 250 °C was maintained for 1 min. The mass spectrometer operated in the electron impact mode with an ionization energy of 70 eV, scanning a range of 35 to 400 a.m.u. Compound identification was performed by comparing the mass spectra and linear retention index (LRI) with those in the NIST 2011 mass spectrum database and available reference standards. The volatile compounds were approximately quantified using the peak area normalization method in the total ion current diagram. All measurements mentioned above were conducted in triplicate for the yeast fermentation broth samples.

#### 3.5.3. Volatile Compounds Detected by GC-IMS

The GC-IMS analysis of volatiles was performed using a FlavourSpec^®^ from G.A.S. (Gesellschaft für Analytische Sensorsysteme mbH, Dortmund, Germany) equipped with a syringe and an autosampler unit for headspace analysis. A 20 mL headspace sample bottle was filled with 3 mL of yeast broth fermented for 72 h and incubated at a constant temperature of 40 °C while being agitated at 500 rpm for 15 min. Subsequently, 500 μL of the headspace content was automatically injected into a heated syringe (85 °C) in a splitless injection mode. Chromatographic separation was accomplished using a WAX-5 capillary column (15 m × 0.53 mm) with nitrogen (N_2_) as the carrier gas (purity ≥ 99.999%) and a column temperature of 60 °C. The programmed inlet gas flow rate was adjusted as follows: 0–2 min, 2 mL/min; 2–5 min, 10 mL/min; 5–15 min, 15 mL/min; 15–20 min, 50 mL/min; 20–30 min, 100 mL/min, with a total GC run time of 30 min. Following separation by a capillary column, the headspace content was introduced into the ionization chamber, then the drift zone, and ultimately the IMS detector. Drift gas (N_2_, purity ≥ 99.999%) flowed at a rate of 150 mL/min. Volatile compounds were identified based on their drift times and retention indices using standards from the NIST and G.A.S. databases. The experiment was conducted in triplicate. All measurements were performed in triplicate.

#### 3.5.4. Calculation of ROAV

The ROAV for each volatile compound was calculated by dividing its concentration by the corresponding odor threshold in water. The methods for determining the odor thresholds in water for each aroma compound and the detailed formulas for calculating the ROAV can be found in work by Xu et al. [11]. A volatile compound with an ROAV greater than 1 is considered an aroma-active compound that contributes to the overall aroma of a sample.

### 3.6. Statistical Analysis

Principal component analyses (PCA) and partial least squares–discriminant analyses (PLS-DA) of volatile profiles were performed in SIMCA software (14.1, Umea, Sweden). The concentrations of volatile compounds were reported as means ± standard deviation (SD). An analysis of variance (ANOVA) was conducted using SPSS software (26.0, IBM, Armonk, NY, USA), considering a *p*-value < 0.05 as indicative of significant differentiation. The chromatograms obtained from GC-IMS were visualized and analyzed utilizing the built-in reporter plug-in of the GC-IMS system. Fingerprint profiles of volatile flavor substances were generated through gallery plot analysis.

## 4. Conclusions

An E-nose, GC-MS, and GC-IMS were employed to rapidly, accurately, and comprehensively characterize the volatile compounds of YB4 in different NaCl concentrations. An E-nose can quickly distinguish differences in volatile substances of YB4 at different NaCl concentrations. Through GC-MS and GC-IMS analyses, it was observed that aldehydes and esters were the primary flavor substances in YB4. In addition, some pyrazines and ketones were identified using GC-IMS. By combining these two analytical methods, rapid identification and comprehensive characterization of the volatile compounds in YB4 can be achieved. The discriminant analysis of YB4 with different NaCl concentrations was carried out by PLS-DA. A total of 35 and 22 difference markers were selected from GC-MS and GC-IMS, which were helpful for the quality evaluation of YB4. Finally, through statistical analysis and confirmation using ROAVs, ten volatiles were identified as primary contributors to the aroma of YB4 at different NaCl concentrations. In summary, based on the analysis results of GC-MS and GC-IMS, YB4 at 6–12% NaCl concentration had an excellent flavor profile, and can be directly used for the subsequent development and application of bioactive agents for aroma-producing yeast. The findings of this study offer valuable insights into the development of yeast agents that produce aroma, thereby promoting the utilization of YB4 in fermented foods beyond just Sichuan pickles. The aforementioned methods can thus be employed for monitoring the alterations in aroma compounds during fermentation and enhancing the quality of saccharomyces fragrans and other fermented food microorganisms.

## Figures and Tables

**Table 1:** The ROAV values of the main volatile compounds by GC-MS.

No.	Volatile Compounds	Odor Description ^#^	Threshold ^ψ^ (μg/L)	CK	APYA	APYB	APYC	APYD
1	Nonanal	Floral, green	3.1	100	100	100	100	100
2	2-methyl-1-propanol	Wine	33	18.14	0.90	27.23	26.45	<0.01
3	3-methyl-1-butanol	Cocoa, floral	6.1	10.01	65.29	<0.01	60.68	86.67
4	Ethanol	Alcohol, floral	620	5.17	0.9	2.26	1.76	1.39
5	1-Pentanol	Fruit, green	153	<0.01	2.61	4.78	3.16	<0.01
6	Phenylethyl alcohol	Floral, fruit	21	<0.01	4.92	<0.01	15.57	<0.01
7	Eucalyptol	Camphor, cool	5.08	<0.01	<0.01	21.60	<0.01	47.57
8	2-methyl-butanal	Almond, cocoa	1	93.89	<0.01	<0.01	<0.01	<0.01
9	Benzeneacetaldehyde	Berry, floral, flower	1.7	<0.01	<0.01	92.86	<0.01	<0.01
10	Octanal	Fruity	170	<0.01	<0.01	3.88	<0.01	<0.01
11	Ethyl butyrate	Apple, banana	126.1	2.80	0.23	5.99	<0.01	<0.01
12	Ethyl isobutyrate	Apple, floral	0.11	57.87	67.13	<0.01	80.70	<0.01
13	Butyl acrylate	—	10	25.48	0.94	7.11	6.64	65.54
14	Isoamyl acetate	Apple, banana, fruit	91.8	<0.01	3.87	<0.01	<0.01	<0.01
15	2-Phenylethyl formate	Rose, green	270	<0.01	<0.01	<0.01	1.08	0.35
16	Acetoin	Butter, cream	14	<0.01	<0.01	<0.01	8.38	<0.01
17	Terpinolene	Pine, plastic, sweet	200	1.54	<0.01	<0.01	<0.01	<0.01
18	β-pinene	Pine, polish	140	2.62	<0.01	<0.01	<0.01	<0.01
19	α-Phellandrene	Citrus, mint, pepper	40	6.08	<0.01	<0.01	<0.01	9.26
20	Limonene	Citrus, fruit	210	1.63	<0.01	0.53	<0.01	<0.01
21	Anethole	Anise, sweet	57	<0.01	1.98	1.72	<0.01	<0.01
22	D-Limonene	Citrus, lemon	45	<0.01	<0.01	<0.01	1.75	<0.01
23	Estragole	Anise, herb	16	4.51	<0.01	<0.01	10.75	29.50
24	N-propylbenzene	Mothball	19	<0.01	1.19	<0.01	2.70	<0.01
25	Palmitic acid ethyl ester	Fatty, fruity, rancid	2000	<0.01	<0.01	<0.01	<0.01	1.17

^#^ CK, APYA, APYB, APYC, and APYD represent YB4 treated with 0%, 6%, 9%, 12%, and 15% NaCl. The odor description was derived from the literature (Flavornet, the odor database). ^ψ^, All odor thresholds were acquired from Odor and Flavor Detection Thresholds in Water (expressed in parts per billion, mg/L). —, no odor description information was found in the literature.

**Table 2:** The ROAV values of the main volatile compounds determined by GC-IMS.

No.	Volatile Compounds	Odor Description ^#^	Threshold ^ψ^ (mg/L)	CK	APYA	APYB	APYC	APYD
1	Ethyl 2-methylpentanoate	Anise, fruit	0.003	100	100	100	100	100
2	Acrolein	Burnt sweet, pungent	8.3	1.24	0.42	0.28	0.27	0.31
3	Ethyl valerate	Apple, herb	0.58	4.11	1.1	0.58	0.52	0.74
4	Ethyl 2-methylbutanoate	Apple, fruit	0.063	5.46	4.29	2.82	2.86	3.55
5	Ethyl isobutanoate	Floral, rubber	0.003	2.21	3.33	12.76	10.45	4.79
6	Ethyl isovalerate	Apple, citrus,	0.11	1.98	0.72	0.62	0.72	1.31
7	Prop-1-ene-3,3’-thiobis	Garlic, pepper	1	6.63	2.80	1.83	1.59	2.05
8	O-Cresol	Must, phenol, smoke	1.2	0.85	1.85	1.56	1.65	1.73
9	2,6-Dimethylphenol	Coffee, phenol	0.2	12.05	26.63	23.81	26.6	26.29
10	2-Acetylpyrazine	Baked, nutty	0.4	1.67	3.87	3.28	3.53	3.43

^#^ CK, APYA, APYB, APYC, and APYD represent YB4 treated with 0%, 6%, 9%, 12%, and 15% NaCl. The odor description was sourced from the literature (Flavornet, the odor database). ^ψ^, All odor thresholds were obtained from Odor and Flavor Detection Thresholds in Water (expressed in parts per billion, mg/L).

## Data Availability

All the data are available within the manuscript.

## Supplementary Materials

The following supporting information can be downloaded at: https://www.mdpi.com/article/10.3390/molecules28196979/s1, Table S1: The information of volatile compounds by GC-MS; Table S2: The information of volatile compounds by GC-IMS.
