# Supplementary material for: Exploring the Profile Contributions in Meyerozyma guilliermondii YB4 under Different NaCl Concentrations Using GC-MS Combined with GC-IMS and an Electronic Nose
Source: Molecules. 2023 Oct 8;28(19):6979. doi: 10.3390/molecules28196979 (PMC10574234; doi:10.3390/molecules28196979)
Supplement: Supplementary file 1 [file molecules-28-06979-s001.zip › molecules-2632063-supplementary.pdf]

Table S1 The information of volatile compounds by GC-MS.

Volatile compounds	CAS	Comparative contant (%)				
		CK	APYA	APYB	APYC	APYD
(R)-(-)-2-HEXANOL	26549-24-6	2.76±0.60[a]	—	—	—	—
(S)-Propane-1,2-diol	4254-15-3	2.84±0.44[a]	—	—	—	—
Isopropyl alcohol	67-63-0	0.34±0.19[a]	0.46±0.10[a]	—	—	—
DL-2,3-Butanediol	6982-25-8	2.53±0.33[a]	—	2.66±0.60[a]	—	5.14±0.30[b]
cis-2-Thujen-4-ol	97631-68-0	0.64±0.26[a]	—	—	—	—
2-Methyl-1-propanol	78-83-1	4.21±0.35[b]	0.48±0.05[a]	4.99±0.90[b]	5.91±0.22[b]	—
3-Methyl-1-butanol	123-51-3	0.43±0.13[a]	6.43±0.82[b]	—	2.51±0.35[b]	2.63±0.30[b]
2-Methyl-1-butanol	137-32-6	10.23±0.60[a]	6.81±0.70[b]	6.57±0.20[b]	8.75±0.47[a]	10.07±0.68[a]
Terpineol	8000-41-7	1.18±0.48[a]	—	4.11±0.92[b]	—	—
Ethanol	64-17-5	22.55±1.22[a]	8.99±0.15[b]	7.79±0.55[bc]	7.39±0.56[c]	4.29±0.24[d]
(R)-(-)-1-Amino-2-propanol	2799-16-8	—	0.61±0.13[a]	—	—	—
1-Pentanol	71-41-0	—	6.43±0.64[a]	4.06±0.78[a]	3.27±0.55[a]	—
1-Butanol	71-36-3	—	1.54±0.15[a]	—	—	—
1-Hexanol	111-27-3	—	0.33±0.03[a]	—	—	—
Phenylethyl alcohol	60-12-8	—	1.67±0.25[a]	—	2.21±0.63[a]	—
L-(+)-Isoleucinol	24629-25-2	—	1.05±0.69[a]	—	—	—
3-Methyl-2-Pentanol	565-60-6	—	3.00±0.63[a]	—	—	—
1-Propanol	71-23-8	—	2.84±0.20[a]	—	—	—

Table S1 (continued)

Volatile compounds	CAS	Comparative contant (%)				
		CK	APYA	APYB	APYC	APYD
2,4-Pentanediol	625-69-4	—	—	1.02±0.21[a]	—	—
D-Leucinol	53448-09-2	—	—	1.66±0.51[a]	—	—
Isopinocarveol	6712-79-4	—	—	5.37±0.56[a]	—	3.57±0.34[b]
2,5-Dimethyl-benzenemethanol	53957-33-8	—	—	0.20±0.05[a]	—	—
Eucalyptol	470-82-6	—	—	0.61±0.81[a]	—	1.20±0.28[b]
3-Methyl-4-Penten-2-ol	1569-59-1	—	—	—	3.48±0.36[a]	—
(R)-(-)-1,2-Propanediol	4254-14-2	—	—	—	0.74±0.20[a]	—
Methyl-benzeneethanol	698-87-3	—	—	—	—	1.46±0.44[a]
(R)-(-)-2-Amino-3-methyl-1-butanol	4276-09-9	—	—	—	—	1.78±0.59[a]
1-Tetracosanol	506-51-4	—	—	—	—	1.18±0.51[a]
1-(2-Methylphenyl)ethanol	7287-82-3	—	—	—	—	1.21±0.36[a]
Nonanal	124-19-6	2.18±0.68[b]	5.00±0.50[a]	1.72±0.10[b]	2.10±0.07[b]	1.54±0.11[b]
Methylglyoxal	78-98-8	0.46±0.40[a]	—	2.98±0.44[b]	—	—
2-Methyl-butanal	96-17-3	0.66±0.27[a]	—	—	—	—
Methoxyacetaldehyde	10312-83-1	—	0.24±0.11[a]	—	—	0.14±0.02[b]
Cuminaldehyde	122-03-2	—	0.60±0.09[a]	—	—	—
5-Methylhexanal	1860-39-5	—	—	0.26±0.03[a]	—	—
Benzeneacetaldehyde	122-78-1	—	—	0.88±0.42[a]	—	—

Table S1 (continued)

Volatile compounds	CAS	Comparative contant (%)				
		CK	APYA	APYB	APYC	APYD
Octanal	124-13-0	—	—	3.66±0.29[a]	—	—
Benzaldehyde	100-52-7	—	—	—	0.49±0.22[a]	—
2-Methyl-3-phenylpropionaldehyde	5445-77-2	—	—	—	0.32±0.21[a]	—
3-Hydroxy-2,2-dimethylpropanal	597-31-9	—	—	—	—	1.21±0.58[b]
Methyltartronic acid	595-98-2	0.20±0.15[a]	0.10±0.05[a]	—	—	0.32±0.13[a]
α-Methyl-DL-valine	26287-62-7	0.18±0.12[a]	—	—	—	—
2,3-Dimethylpentanoic acid	82608-03-5	—	0.75±0.58[a]	—	—	—
D-Alanine	338-69-2	—	—	0.63±0.21[a]	—	—
Methoxyacetic acid	625-45-6	—	—	0.59±0.12[a]	—	—
Hydroxyacetic acid	79-14-1	—	—	—	0.14±0.05[a]	—
4-Carboxycyclohexanone	874-61-3	—	—	—	2.08±0.23[a]	—
Ethyl propionate	105-37-3	1.70±0.62[a]	5.22±1.41[b]	2.67±1.20[a]	5.30±1.13[b]	—
Ethyl butyrate	105-54-4	2.48±0.25[b]	0.48±0.12[a]	4.19±0.33[b]	—	—
Isopentyl formate	110-45-2	0.78±0.26[a]	—	—	—	—
Ethyl Acetate	141-78-6	2.61±0.36[d]	8.58±0.53[b]	14.09±0.66[a]	15.36±0.34[a]	4.95±0.92[c]
Pentanoic acid, 5-hydroxy-, 2,4-bis(1,1-dimethylethyl)phenyl ester	166273-38-7	0.15±0.03[a]	—	—	—	—
Ethyl 8-bromooctanoate	29823-21-0	0.07±0.02[a]	—	—	—	—
Ethyl alaninate	3082-75-5	1.26±0.44[a]	—	—	—	6.21±0.75[b]

Table S1 (continued)

Volatile compounds	CAS	Comparative contant (%)				
		CK	APYA	APYB	APYC	APYD
Ethanedioic acid, MonoMethyl ester	600-23-7	2.59±1.27[a]	—	5.94±0.42[a]	—	—
Ethyl isobutyrate	97-62-1	0.04±0.00[a]	0.96±1.41[b]	—	0.06±0.02[ab]	—
Methyl formate	107-31-3	1.65±0.92[a]	—	—	—	—
Butyl acrylate	141-32-2	1.96±0.56[a]	0.15±0.04[b]	0.39±0.44[b]	0.45±0.37[b]	3.26±0.25[a]
Ethyl Hexanoate	123-66-0	—	3.76±0.44[a]	—	—	—
Isobutyl acetate	110-19-0	—	0.11±0.02[a]	0.35±0.42[ab]	1.19±0.20[b]	—
Propyl propionate	106-36-5	—	0.17±0.05[a]	—	—	—
Ethyl 3-phenylpropionate	2021-28-5	—	0.27±0.14[a]	—	—	—
2,2-Dimethyl-1,3-propanediol dinitrate	26482-65-5	—	0.64±0.34[a]	—	—	—
Ethyl (2Z)-2-methoxyimino-3-oxo-butanoate	60846-14-2	—	1.67±0.51[a]	—	1.38±0.35[a]	—
2-Hydroxyethyl formate	628-35-3	—	1.94±0.67[a]	—	—	—
Isoamyl lactate	19329-89-6	—	4.85±0.76[a]	—	—	—
Isoamyl acetate	123-92-2	—	5.74±0.07[a]	—	—	—
Methyl 2-oxoacetate	922-68-9	—	0.68±0.12[a]	—	4.90±0.51[b]	—
Ethyl nitrite	109-95-5	—	—	1.96±0.27[a]	—	—
Amyl acetate	628-63-7	—	—	3.33±0.86[a]	—	—
2-Phenylethyl formate	104-62-1	—	—	—	3.54±0.86[a]	0.47±0.26[b]
Ethyl 10-BroModecanoate	55099-31-5	—	—	—	3.29±0.65[a]	—

Table S1 (continued)

Volatile compounds	CAS	Comparative contant (%)				
		CK	APYA	APYB	APYC	APYD
Isocyanatomethane	624-83-9	—	—	—	2.37±0.48[a]	—
Butyl formate	592-84-7	—	—	—	2.91±0.74[a]	—
1,2-Propanediol dinitrate	6423-43-4	—	—	—	—	4.33±0.64[a]
ethyl methyl(nitroso)carbamate	615-53-2	—	—	—	—	2.17±0.25[a]
Palmitic acid ethyl ester	628-97-7	—	—	—	—	11.65±0.52[a]
Xanthoxylin	90-24-4	0.63±0.27[a]	—	—	—	—
Piperonyl acetone	55418-52-5	—	1.12±0.04[a]	—	—	—
4'-Methylvalerophenone	1671-77-8	—	—	—	1.62±0.39[a]	—
4-Acetoxy-2-azetidinone	28562-53-0	—	—	—	1.91±0.26[a]	—
Acetoin	513-86-0	—	—	—	0.79±0.29[a]	—
Methoxyacetone	5878-19-3	—	—	—	0.36±0.22[a]	—
4-Hydroxy-2-butanone	590-90-9	—	—	—	2.86±0.22[a]	—
2-Propanone,1-(1-methylethoxy)-	42781-12-4	—	—	—	—	1.88±0.86[a]
(R)-1-methyl-5-(1-methylvinyl)cyclohexene	1461-27-4	0.11±0.01[a]	1.22±0.37[b]	—	—	—
Spiro[3.3]hepta-1,5-diene	22635-78-5	0.04±0.03[a]	—	—	—	—
(Z)-Anethole	25679-28-1	1.47±0.78[a]	—	—	0.69±0.27[a]	—
4-Methoxybut-1-ene	4696-30-4	0.80±0.12[a]	—	—	—	—
Undecane,5-methylene-	5698-48-6	0.10±0.04[a]	—	—	—	—

Table S1 (continued)

Volatile compounds	CAS	Comparative contant (%)				
		CK	APYA	APYB	APYC	APYD
α-Terpinene	99-86-5	2.43±0.08[a]	—	—	—	—
Terpinolene	586-62-9	2.16±0.32[a]	—	—	—	—
2-methyl-6-methylene-1,7-Octadiene	1686-30-2	1.22±0.23[a]	—	—	—	—
Ocimene	13877-91-3	2.18±0.22[a]	—	—	—	—
β-Pinene	127-91-3	2.58±0.64[a]	—	—	—	—
α-Phellandrene	99-83-2	1.71±0.30[a]	—	—	—	1.84±0.15[a]
Carene	13466-78-9	1.67±0.06[a]	—	0.33±0.24[a]	—	—
Limonene	138-86-3	2.40±0.74[a]	—	0.62±0.34[a]	—	—
α-Elemene	33880-83-0	0.30±0.00[a]	—	—	—	—
2,4-Dimethylstyrened	2234-20-0	1.28±0.20[a]	—	—	—	—
(E)-5-Methyl-4-decene	62338-51-6	0.17±0.09[a]	—	—	—	—
Anethole	104-46-1	—	1.82±0.65[a]	0.54±0.17[a]	—	—
7-Ethyl-1,3,5-cycloheptatriene	17634-51-4	—	—	0.02±0.01[a]	—	—
Cyclohexene, 1-methyl-5-(1-methylethenyl)-	13898-73-2	—	—	0.70±0.03[a]	—	1.07±0.11[b]
D-Limonene	5989-27-5	—	—	—	0.53±0.21[a]	—
(3E)-3-prop-2-enylidenecyclobutene	52097-85-5	—	—	—	—	2.21±0.21[a]
p-Cymene	99-87-6	—	—	—	—	0.20±0.12[a]
Trans-2,4-dimethyloxetane	29424-94-0	0.60±0.27[a]	—	—	—	—

Table S1 (continued)

Volatile compounds	CAS	Comparative contant (%)				
		CK	APYA	APYB	APYC	APYD
2,3-Epoxy-4,4-dimethylpentane	53897-30-6	3.48±0.93[a]	—	2.45±0.53[a]	—	—
(2R,3R)-rel-Oxirane,2,3-bis(1-methylethyl)	54644-32-5	0.06±0.02[a]	—	—	—	—
1,4-Bis(phenylmethyl)-2,3,5-trioxabicyclo[2.1.0]pentane	56247-48-4	—	—	0.20±0.08[a]	—	—
3-Methoxyhexane	54658-01-4	—	—	1.22±0.09[a]	—	—
7-Hexylicosane	55333-99-8	—	—	2.17±0.37[a]	—	—
9-Hexylheptadecane	55124-79-3	—	—	0.92±0.14[a]	—	—
Decane	124-18-5	—	—	—	0.60±0.33[a]	—
2-Butene oxide	3266-23-7	—	—	—	1.93±0.58[a]	—
2,3,5-Trimethyldecane	62238-11-3	—	—	—	0.85±0.18[a]	—
1,3-diphenylpropane	1081-75-0	—	—	—	—	0.44±0.25[a]
sec-Butylamine	13952-84-6	—	—	—	—	0.76±0.32[a]
Decane	124-18-5	—	—	—	—	2.17±0.54[a]
Tetratriacontane	14167-59-0	—	—	—	—	3.98±0.25[a]
1,54-dibromo-Tetrapentacontane	852228-22-9	—	—	—	—	2.17±0.13[a]
Tetracosane	646-31-1	—	—	—	—	1.35±0.35[a]
3-Methyl-3-phenyl-azetidine	5961-33-1	—	—	—	—	0.26±0.18[a]
2,4-Di-tert-butylphenol	96-76-4	—	0.69±0.23[a]	0.58±0.44[a]	1.08±0.15[a]	2.43±0.55[a]
2,6-Di-tert-butylphenol	128-39-2	—	—	—	0.22±0.11[a]	—

Table S1 (continued)

Volatile compounds	CAS	Comparative contant (%)				
		CK	APYA	APYB	APYC	APYD
Estragole	140-67-0	0.51±0.15[a]	—	—	1.16±0.19[a]	2.35±0.47[a]
2-Methoxyethanol	109-86-4	—	5.49±0.71[a]	—	—	0.32±0.24[a]
N-butyl methyl ether	628-28-4	—	—	0.28±0.21[a]	—	—
Ethyl methyl ether	540-67-0	—	—	1.71±0.33[a]	—	—
2-Butoxyethanol	111-76-2	0.15±0.05[a]	—	0.26±0.16[a]	—	0.97±0.13[a]
Dimethyl ether	115-10-6	—	—	—	—	0.09±0.04[a]
2-Ethoxyethylamine	110-76-9	0.14±0.07[c]	0.02±0.01[a]	—	0.63±0.21[c]	0.75±0.16[c]
Carbon dioxide	124-38-9	1.59±0.44[b]	2.00±1.05[b]	—	—	2.49±0.89[b]
1-Ethyl-2,4-dimethyl-Benzene	874-41-9	0.51±0.25[b]	—	—	—	0.17±0.10[b]
Nitrogen dioxide	10102-44-0	2.88±0.64[b]	2.63±0.29[b]	0.63±0.33[b]	—	—
Di-tert-butyl peroxide	110-05-4	1.33±0.21[c]	—	1.27±0.18[c]	—	0.11±0.03[b]
N-PropylBenzene	103-65-1	—	0.37±0.18[a]	—	0.35±0.27[a]	—
2,5-Dimethylfuran	625-86-5	—	0.33±0.25[a]	—	—	—
2-PhenylethylaMine	64-04-0	—	0.04±0.02[a]	—	—	—
Tetramethylene diisocyanate	4538-37-8	—	0.06±0.01[a]	—	—	—
(Methoxymethoxymethyl)benzene	31600-55-2	—	—	0.16±0.09[a]	—	—
2-Hexanamine	5329-79-3	—	—	0.06±0.03[a]	—	—
5-Amino-1,3-diphenyl-Pyrazole	5356-71-8	—	—	0.29±0.17[a]	—	—

Table S1 (continued)

Volatile compounds	CAS	Comparative contant (%)				
		CK	APYA	APYB	APYC	APYD
Urea	57-13-6	—	—	—	1.08±0.16[a]	—
Ammonium acetate	631-61-8	—	—	—	0.09±0.02[a]	—
Oxiranemethanol, 2-phenyl-	141248-89-7	—	—	—	—	0.03±0.02[a]

The term"—"means the compound was not detected in sample; Different letters in the same column indicated significant differences ($p < 0.05$).

Table S2 The information of volatile compounds by GC-IMS

Volatile Compounds	CAS	RI	RT	DT	Comparative contant (%)				
					CK	APYA	APYB	APYC	APYD
2-ethyl-1-hexanol	104-76-7	1028.7	391.882	1.38717	6.21±0.19[b]	7.15±0.12[a]	7.59±0.04[a]	7.18±0.37[a]	7.52±0.21[a]
2-methyl-1-propanol-M	78-83-1	1073.5	475.509	1.17871	0.77±0.01[a]	0.59±0.01[b]	0.51±0.01[c]	0.52±0.01[c]	0.59±0.02[b]
1-Propanol	71-23-8	1031.7	397.4	1.25042	0.37±0.01[c]	0.45±0.02[b]	0.49±0.01[ab]	0.52±0.02[a]	0.50±0.02[a]
2-furanmethanol	98-00-0	888.5	239.797	1.13529	0.17±0.00[a]	0.11±0.01[b]	0.10±0.00[b]	0.10±0.01[b]	0.12±0.01[b]
2-Hexen-1-ol	2305-21-7	863.8	225.608	1.2015	0.07±0.00[b]	0.06±0.00[c]	0.07±0.00[b]	0.07±0.00[b]	0.10±0.00[a]
2-Methyl propanol-D	78-83-1	1073.7	475.833	1.38399	0.14±0.01[c]	0.49±0.01[b]	0.52±0.01[b]	0.67±0.01[a]	0.52±0.03[b]
(Z)3-Octen-1-ol-M	20125-84-2	1054	439.106	1.30605	0.04±0.00[e]	0.16±0.00[d]	0.22±0.00[c]	0.29±0.01[a]	0.26±0.01[b]
(Z)3-Octen-1-ol-D	20125-84-2	1053.3	437.679	1.75153	0.04±0.01[d]	0.09±0.01[c]	0.27±0.01[b]	0.58±0.02[a]	0.30±0.01[b]
(Z)-4-heptenal	6728-31-0	883.7	237.029	1.59176	0.05±0.00[a]	0.04±0.00[a]	0.05±0.00[a]	0.05±0.01[a]	0.04±0.00[a]
Ethanol	64-17-5	970.6	309.552	1.03854	8.86±0.12[a]	2.51±0.09[b]	2.12±0.05[c]	1.90±0.07[d]	2.48±0.04[b]
3-(methylsulfanyl)propanol (methionol)	505-10-2	978.8	316.57	1.47376	0.02±0.00[e]	0.04±0.00[c]	0.06±0.00[a]	0.05±0.00[b]	0.04±0.00[d]
2-propanol	67-63-0	891.5	241.535	1.09787	0.44±0.01[a]	0.11±0.01[c]	0.11±0.00[c]	0.11±0.01[c]	0.14±0.00[b]
Butan-2-ol	78-92-2	1026.7	388.222	1.17329	0.48±0.01[a]	0.16±0.01[b]	0.13±0.00[cd]	0.12±0.01[d]	0.15±0.01[bc]
(E,E)-2,4-Hexadienal	142-83-6	1410.9	1104.574	1.11275	0.14±0.01[b]	0.21±0.00[a]	0.05±0.00[c]	0.04±0.00[c]	0.04±0.00[c]
2-Methyl-2-pentenal	623-36-9	1149.4	616.988	1.50178	7.01±0.25[e]	13.92±0.13[d]	15.75±0.04[c]	16.29±0.11[b]	16.79±0.11[a]
Heptanal	111-71-7	1159.1	634.992	1.32241	0.51±0.02[b]	1.07±0.04[a]	1.05±0.03[a]	1.00±0.01[a]	1.05±0.02[a]
Hexanal	66-25-1	1072.6	473.717	1.26055	0.20±0.01[d]	0.70±0.02[c]	0.77±0.00[b]	0.80±0.00[a]	0.76±0.01[b]

Table S2 (continued)

Volatile Compounds	CAS	RI	RT	DT	Comparative contant (%)				
					CK	APYA	APYB	APYC	APYD
Pentanal	110-62-3	984.3	321.321	1.19327	0.24±0.04^{a}	0.08±0.00^{b}	0.08±0.01^{b}	0.10±0.05^{b}	0.10±0.02^{b}
(E)-2-hexenal	6728-26-3	852.3	219.004	1.19935	0.22±0.01^{a}	0.16±0.01^{b}	0.22±0.01^{a}	0.18±0.03^{ab}	0.22±0.01^{a}
Butanal	123-72-8	850.6	217.986	1.27854	0.20±0.01^{b}	0.14±0.01^{c}	0.21±0.01^{b}	0.14±0.03^{c}	0.27±0.02^{a}
Octanal	124-13-0	999.1	336.711	1.39011	0.08±0.01^{d}	0.27±0.01^{c}	0.37±0.01^{b}	0.68±0.05^{a}	0.66±0.01^{a}
acrolein	107-02-8	875.3	232.182	1.0003	6.28±0.09^{a}	3.66±0.25^{b}	3.35±0.08^{bc}	3.10±0.25^{c}	3.20±0.05^{c}
isobutyl butyrate	539-90-2	1148.5	615.236	1.78982	0.10±0.01^{d}	0.39±0.02^{c}	0.47±0.01^{b}	0.52±0.01^{a}	0.48±0.02^{b}
Butyl acetate	123-86-4	1114.9	552.66	1.25645	0.30±0.02^{a}	0.10±0.00^{b}	0.10±0.00^{b}	0.10±0.00^{b}	0.10±0.01^{b}
Isoamyl acetate	123-92-2	1141.4	602.104	1.31224	0.29±0.00^{c}	0.55±0.01^{b}	0.58±0.02^{a}	0.56±0.01^{ab}	0.56±0.00^{ab}
ethyl valerate	539-82-2	1142.3	603.638	1.2386	1.45±0.04^{a}	0.67±0.02^{b}	0.48±0.01^{cd}	0.42±0.04^{d}	0.53±0.03^{c}
lsovaleric acid, isobutyl ester	589-59-3	1007.7	352.761	1.89185	0.45±0.05^{a}	0.38±0.03^{a}	0.40±0.01^{a}	0.38±0.09^{a}	0.42±0.04^{a}
methyl hexanoate	106-70-7	907.5	255.118	1.6846	4.37±0.06^{a}	2.34±0.04^{bc}	2.10±0.15^{c}	2.22±0.32^{c}	2.70±0.15^{b}
Ethyl 2-methylbutanoate	7452-79-1	1026.3	387.39	1.2542	0.21±0.01^{b}	0.28±0.01^{a}	0.25±0.00^{a}	0.25±0.02^{a}	0.27±0.01^{a}
Ethyl acetate	141-78-6	895.1	244.386	1.33695	2.26±0.07^{c}	5.16±0.03^{a}	5.34±0.08^{a}	5.14±0.16^{a}	4.83±0.03^{b}
Methyl heptanoate	106-73-0	1007.1	351.531	1.80383	2.98±0.33^{a}	2.81±0.19^{a}	3.26±0.15^{a}	3.34±0.87^{a}	3.73±0.43^{a}
Ethyl formate	109-94-4	841.7	212.871	1.06962	1.00±0.04^{a}	0.82±0.06^{b}	0.79±0.01^{bc}	0.74±0.01^{c}	0.79±0.01^{b}
Bornylacetate	76-49-3	1282.1	864.456	1.23675	1.43±0.02^{a}	1.00±0.05^{b}	0.53±0.03^{c}	0.52±0.01^{c}	0.55±0.02^{c}
Ethyl isobutanoate	97-62-1	940.9	283.877	1.56482	0.15±0.00^{e}	0.38±0.02^{d}	1.99±0.05^{a}	1.59±0.13^{b}	0.65±0.03^{c}
Methyl acrylate	96-33-3	934.9	278.749	1.26771	0.07±0.01^{c}	0.19±0.00^{b}	0.27±0.00^{a}	0.27±0.01^{a}	0.27±0.00^{a}

Table S2 (continued)

Volatile Compounds	CAS	RI	RT	DT	Comparative contant (%)				
					CK	APYA	APYB	APYC	APYD
5-ethyldihydro-2(3h)-furanone	695-06-7	1052.9	437.007	1.53814	0.04±0.00^{d}	0.09±0.00^{c}	0.17±0.01^{b}	0.21±0.00^{a}	0.17±0.01^{b}
Isoamyl butyrate	106-27-4	1053.3	437.77	1.40915	0.03±0.01^{d}	0.18±0.01^{c}	0.30±0.01^{b}	0.41±0.00^{a}	0.30±0.00^{b}
Methyl 3-(methylthio)propanoate	13532-18-8	1029.5	393.383	1.60547	0.10±0.00^{c}	0.11±0.01^{b}	0.13±0.00^{a}	0.13±0.01^{ab}	0.11±0.00^{b}
Ethyl propanoate	105-37-3	934.5	278.377	1.458	0.03±0.00^{e}	0.12±0.00^{d}	0.65±0.01^{b}	0.72±0.05^{a}	0.38±0.01^{c}
Isobutyl formate	542-55-2	937	280.559	1.50946	0.03±0.00^{d}	0.09±0.01^{c}	0.41±0.01^{a}	0.39±0.03^{a}	0.20±0.00^{b}
Propyl acetate	109-60-4	947.4	289.49	1.47999	0.01±0.00^{d}	0.08±0.01^{a}	0.05±0.00^{b}	0.07±0.01^{ab}	0.03±0.00^{c}
Methyl butanoate	623-42-7	998.7	335.89	1.43251	0.02±0.00^{d}	0.05±0.00^{b}	0.05±0.00^{a}	0.05±0.00^{b}	0.04±0.00^{c}
Amyl acetate	628-63-7	912.2	259.136	1.33657	0.27±0.01^{a}	0.24±0.01^{a}	0.28±0.02^{a}	0.24±0.05^{a}	0.22±0.01^{a}
Ethyl lactate	97-64-3	814	196.986	1.13274	0.27±0.01^{c}	0.54±0.01^{b}	0.62±0.00^{a}	0.57±0.01^{b}	0.61±0.02^{a}
Iso-Propyl acetate	108-21-4	862.4	224.785	1.1557	0.02±0.00^{c}	0.12±0.00^{a}	0.12±0.00^{a}	0.13±0.01^{a}	0.08±0.00^{b}
Ethyl 2-methylpentanoate	39255-32-8	941.3	284.266	1.31773	0.18±0.00^{d}	0.31±0.00^{c}	0.43±0.01^{a}	0.42±0.00^{a}	0.37±0.01^{b}
(Z)-3-Hexenyl propionate	33467-74-2	1113.1	549.33	1.36241	0.09±0.00^{a}	0.06±0.00^{b}	0.03±0.00^{c}	0.03±0.00^{c}	0.03±0.00^{c}
Ethyl 3-hydroxyhexanoate	2305-25-1	1128.8	578.513	1.30313	0.18±0.00^{a}	0.07±0.00^{b}	0.07±0.00^{b}	0.07±0.02^{b}	0.08±0.01^{b}
Ethyl isovalerate	108-64-5	850.2	217.795	1.67356	0.13±0.03^{ab}	0.08±0.01^{b}	0.10±0.01^{b}	0.11±0.03^{ab}	0.18±0.02^{a}
2-Methylbutanoic acid	116-53-0	846.1	215.416	1.47197	0.82±0.14^{a}	0.60±0.03^{a}	0.52±0.06^{a}	0.65±0.23^{a}	0.74±0.09^{a}
Prop-1-ene-3,3'-thiobis	592-88-1	857.2	221.814	1.11416	4.04±0.11^{a}	2.92±0.02^{b}	2.61±0.05^{c}	2.21±0.09^{d}	2.52±0.01^{c}
o-Cresol	95-48-7	1061.9	453.779	1.13528	0.62±0.02^{d}	2.32±0.06^{c}	2.66±0.08^{ab}	2.74±0.03^{a}	2.55±0.06^{b}
2,6-dimethylphenol	576-26-1	1103.2	530.897	1.13423	1.47±0.08^{d}	5.56±0.16^{c}	6.76±0.18^{b}	7.38±0.23^{a}	6.45±0.07^{b}

Table S2 (continued)

Volatile Compounds	CAS	RI	RT	DT	Comparative contant (%)				
					CK	APYA	APYB	APYC	APYD
Octan-2-one	111-13-7	1282.6	865.388	1.33588	23.14±0.22^{a}	16.23±0.18^{b}	9.18±0.26^{d}	9.50±0.22^{d}	10.11±0.25^{c}
Acetoin	513-86-0	1273.1	847.64	1.06799	1.40±0.01^{a}	0.71±0.01^{b}	0.58±0.01^{d}	0.56±0.01^{d}	0.65±0.01^{c}
MIBK	108-10-1	1032.6	399.137	1.17	1.43±0.01^{a}	0.71±0.02^{bc}	0.67±0.01^{c}	0.62±0.02^{d}	0.73±0.02^{b}
Butan-2-one	78-93-3	953	294.295	1.23543	0.31±0.02^{a}	0.20±0.00^{d}	0.22±0.00^{cd}	0.22±0.01^{c}	0.25±0.00^{b}
Acetone	67-64-1	842.5	213.362	1.14547	0.65±0.03^{c}	0.82±0.04^{a}	0.78±0.03^{ab}	0.72±0.05^{bc}	0.64±0.03^{c}
Pentan-2-one	107-87-9	1002.8	343.621	1.1188	1.00±0.01^{a}	0.82±0.01^{b}	0.79±0.01^{b}	0.74±0.03^{c}	0.79±0.00^{b}
3-Pentanone	96-22-0	976.2	314.311	1.34025	0.06±0.01^{d}	0.39±0.01^{c}	0.48±0.01^{a}	0.43±0.02^{b}	0.35±0.01^{c}
2-Cyclohexen-1-one	930-68-7	935.3	279.089	1.39625	0.02±0.00^{d}	0.05±0.00^{c}	0.12±0.00^{a}	0.12±0.00^{a}	0.07±0.00^{b}
2-Heptanone	110-43-0	898.9	247.661	1.63906	0.31±0.01^{a}	0.06±0.00^{c}	0.06±0.01^{c}	0.06±0.01^{c}	0.09±0.01^{b}
d-Camphor	464-49-3	1127.8	576.683	1.34695	0.10±0.00^{a}	0.10±0.00^{a}	0.11±0.01^{a}	0.12±0.02^{a}	0.11±0.01^{a}
Styrene	10042-5	1293.3	885.253	1.07016	3.69±0.12^{a}	1.92±0.04^{b}	1.48±0.05^{c}	1.34±0.05^{c}	1.50±0.01^{c}
Limonene	138-86-3	1197.6	706.903	1.21548	0.13±0.00^{a}	0.08±0.01^{b}	0.09±0.00^{b}	0.09±0.02^{b}	0.10±0.02^{ab}
β-pinene	127-91-3	1128	576.989	1.21578	0.32±0.00^{a}	0.19±0.01^{b}	0.17±0.01^{b}	0.18±0.06^{b}	0.17±0.02^{b}
α-pinene	80-56-8	998.6	335.669	1.26391	2.66±0.01^{d}	3.62±0.03^{a}	3.55±0.04^{a}	3.19±0.06^{b}	2.96±0.03^{c}
β-myrcene	123-35-3	977.3	315.265	1.61372	0.02±0.00^{d}	0.35±0.03^{b}	0.57±0.04^{a}	0.41±0.04^{b}	0.25±0.01^{c}
2,2,4,6,6-pentamethylheptane	105-57-7	901.6	250.055	0.99546	0.08±0.01^{a}	0.04±0.00^{d}	0.05±0.00^{c}	0.05±0.00^{c}	0.07±0.00^{b}
Diethyl acetal	13475-82-6	951.8	293.269	1.37348	1.03±0.04^{a}	0.22±0.02^{b}	0.18±0.02^{b}	0.15±0.05^{b}	0.22±0.03^{b}
2-Ethyl-5-methylpyrazine	13360-64-0	1001.4	340.968	1.20206	0.47±0.01^{a}	0.43±0.02^{ab}	0.41±0.01^{b}	0.36±0.02^{c}	0.45±0.02^{a}

Table S2 (continued)

Volatile Compounds	CAS	RI	RT	DT	Comparative contant (%)				
					CK	APYA	APYB	APYC	APYD
2-Methoxy-3-methylpyrazine	63450-30-6	976.4	314.49	1.54699	0.01±0.00[c]	0.04±0.00[b]	0.05±0.00[a]	0.04±0.00[b]	0.04±0.00[b]
2-Acetylpyrazine	22047-25-2	1019.1	374.062	1.13076	0.41±0.03[b]	1.62±0.09[a]	1.87±0.05[a]	1.96±0.38[a]	1.68±0.15[a]
2-Ethylpyrazine	13925-00-3	946.4	288.61	1.52029	0.01±0.00[d]	0.05±0.00[c]	0.15±0.00[a]	0.14±0.01[b]	0.05±0.00[c]
2-Ethyl-6-methylpyrazine	13925-03-6	997.1	332.873	1.19597	0.35±0.00[c]	0.52±0.01[ab]	0.53±0.00[a]	0.50±0.02[b]	0.50±0.01[ab]
N-Nitrosodibutylamine	924-16-3	1703.1	1649.527	1.41582	0.42±0.02[d]	0.67±0.02[c]	0.92±0.02[b]	0.86±0.02[b]	1.29±0.06[a]
3-Ethyl-pyridine	536-78-7	1382.1	1050.893	1.11275	0.45±0.02[a]	0.20±0.00[b]	0.09±0.01[c]	0.10±0.00[c]	0.10±0.01[c]
Benzothiazole	95-16-9	1216.1	741.35	1.1773	0.22±0.01[d]	0.32±0.01[b]	0.35±0.01[a]	0.27±0.01[c]	0.28±0.01[c]
Pyridine	110-86-1	1157.1	631.28	1.24446	2.00±0.03[a]	0.92±0.00[b]	0.86±0.00[c]	0.80±0.02[d]	0.92±0.02[b]
2,4,5-Trimethylthiazole	13623-11-5	997.7	334.148	1.55844	0.06±0.00[c]	0.04±0.00[d]	0.04±0.00[d]	0.17±0.03[b]	0.29±0.02[a]
2-Ethylfuran	3208-16-0	947.9	289.942	1.27987	0.04±0.01[c]	0.30±0.01[a]	0.29±0.01[a]	0.30±0.01[a]	0.21±0.01[b]
5,6,7,8-tetrahydroquinoxaline	34413-35-9	1275	851.229	1.20521	0.45±0.01[b]	0.52±0.02[a]	0.45±0.02[b]	0.47±0.01[b]	0.47±0.01[b]
Isoquinoline	119-65-3	1289.5	878.204	1.20383	0.57±0.00[b]	0.61±0.03[b]	0.81±0.02[a]	0.82±0.02[a]	0.82±0.00[a]
2-Pentyl furan	3777-69-3	977.9	315.821	1.23409	0.06±0.01[c]	0.14±0.01[a]	0.14±0.00[a]	0.12±0.00[b]	0.13±0.01[ab]
4-Methyl-5-vinylthiazole	1759-28-0	1042.7	418.077	1.13109	0.62±0.01[d]	2.00±0.06[c]	2.28±0.08[b]	2.46±0.07[a]	2.24±0.06[b]
3-Butenenitrile	109-75-1	1195.9	703.697	1.13528	1.53±0.15[c]	4.32±0.26[b]	5.07±0.07[a]	5.12±0.06[a]	4.68±0.02[b]

* Notes: MW—molecular mass; RI—retention index; Rt—retention time; Dt—drift time. Dimers formed in the IMS drift tube were represented by symbol -D ; Monomers were represented by symbol -M; Different letters in the same row indicated significant differences (p <0.05)
